# Exploring the Challenges of Using Minimal Invasive Surgery to Treat Stress Urinary Incontinence: Insights from a Retrospective Case-Control Study

**DOI:** 10.3390/diagnostics14030323

**Published:** 2024-02-02

**Authors:** Adrian Hașegan, Ionela Mihai, Cosmin Adrian Teodoru, Ioana Bogdan Matacuta, Horațiu Dura, Samuel Bogdan Todor, Cristian Ichim, Denisa Tanasescu, Nicolae Grigore, Ciprian Nicolae Bolca, Cosmin Ioan Mohor, Călin Ilie Mohor, Nicolae Bacalbașa, Dan Georgian Bratu, Adrian Boicean

**Affiliations:** 1 Faculty of Medicine, Lucian Blaga University of Sibiu, 550169 Sibiu, Romania; adrian.hasegan@ulbsibiu.ro (A.H.); ionela.mihai@ulbsibiu.ro (I.M.); adrian.teodoru@ulbsibiu.ro (C.A.T.); ioana.matacutabogdan@ulbsibiu.ro (I.B.M.); horatiu.dura@ulbsibiu.ro (H.D.); samuelbogdant@gmail.com (S.B.T.); cristian.ichim@ulbsibiu.ro (C.I.); denisa.tanasescu@ulbsibiu.ro (D.T.); nicolae.grigore@ulbsibiu.ro (N.G.); cosmin.mohor@ulbsibiu.ro (C.I.M.); calin.mohor@ulbsibiu.ro (C.I.M.); adrian.boicean@ulbsibiu.ro (A.B.); 2 Surgery Department, Universite de Sherbrooke, Sherbrooke, QC J1K 2R1, Canada; ciprian.nicolae.bolca@usherbrooke.ca; 3 Surgery Department, University of Medicine and Pharmacy “Carol Davila” Bucharest, 020021 Bucharest, Romania; nicolaebacalbasa@gmail.com

**Keywords:** stress urinary incontinence, surgery, transobturator tape, tension-free vaginal tape

## Abstract

Stress urinary incontinence (SUI) is a significant global health issue that particularly affects females, leads to notable societal and economic challenges and significantly affects the quality of life. This study focuses on the comparative analysis of two established surgical interventions, tension-free vaginal tape (TVT) and transobturator tape (TOT), at a single center and applied to 455 women suffering from SUI, with a mean follow-up period of 102 ± 30 months for TVT and 80.4 ± 13 months for TOT. Our findings indicate that, in comparison to TVT, the TOT procedure demonstrates fewer early and late post-operative complications in patient outcomes (1.41% vs. 17.64% and; 5.66% vs. 12.74%, both respectively). However, the TVT procedure shows a modestly favorable outcome in the risk of recurrence of SUI, compared to TOT (0% vs. 3.7%); the TOT procedure has also proven to be more effective in alleviating of urgency symptoms, although not at a statistically significant level (*p* = 0.072). Univariable and multivariable analysis of factors that predict late complications showed that only obesity can predict a worse outcome [OR]: 1.125 CI 95%: 1.105–1.533, *p* = 0.037), when adjustments are made for symptoms presented before surgery and procedure type. While both methods are safe and effective, the choice between them should be based on the specific characteristics of each case.

## 1. Introduction

Stress urinary incontinence (SUI) is characterized by the unintentional leakage of urine during moments of physical exertion, such as coughing or sneezing. This condition, a prevalent public health issue, impacts one out of every three women, leading to a substantial decline in their quality of life. It creates a range of challenges, including social, psychological, occupational, physical and sexual difficulties, in those affected [1,2,3,4].

While treatment options such as suburethral tape fixation in a retropubic manner–tension-free vaginal-tape (TVT) or transobturator tape (TOT) have been increasingly used in the last decade, there are considerable possible complications associated with them [5].

TVT is a minimally invasive standard procedure that has been used in the treatment of SUI since 1996, when it was first described by Ulmsten. Since then, an impressive number of procedures have been carried out, yielding mid-term results comparable to colposuspension, with few reported complications. However, described complications included damage to the bladder, intestine and large blood vessels, infections, and difficulties in urinary evacuation and urinary urgency [6,7,8,9,10].

In 2001, Delorme described a new method of tape application, which passes through the obturator foramen, avoiding some complications. In this technique, after the incision of the anterior vaginal wall and the dissection of the intervesicovaginal space, the ischiopubic branch is palpated and the site of the integument incision is marked with the fitting of the suburethral mesh, from outside to inside. Through this technique, Delorme demonstrated that the success rate is higher, with a lower rate of complications, when there are no bladder perforations [11].

Polypropylene strips, prostheses, or meshes refer to synthetic materials available in various shapes and sizes, which are specifically crafted to repair or replac anatomical defects [12]. Polypropylene strips are used in SUI surgery, as an alternative to autologous fascia, with the aim of reducing intervention time, postoperative pain, length of hospital stay and, at least theoretically, increasing durability and improving postoperative results [13,14,15]. For ideal material in these medical procedures, durability stands as a key attribute [16,17]. It should maintain its integrity without, and risk of fracturing or deteriorating, unlike autologous fascia, and must be flexible, robust, hypoallergenic and be seamlessly integrated with surrounding tissues. [13].

The intraoperative complications associated with the two surgical techniques used in our study are bladder perforations, vaginal perforations, and significant bleeding. Postoperative complications, meanwhile, are urinary infections, acute or chronic urinary retention, vaginal erosion, de novo urgency, dysuria, sexual dysfunction and, recurrence of urinary incontinence. In the literature, the complication rate of the two surgical techniques is reported to be low [8,18].

The objective of the paper is to analyze the risk of both intraoperative and postoperative complications arising when TOT and TVT procedures are applied.

## 2. Materials and Method

**Figure 1:**
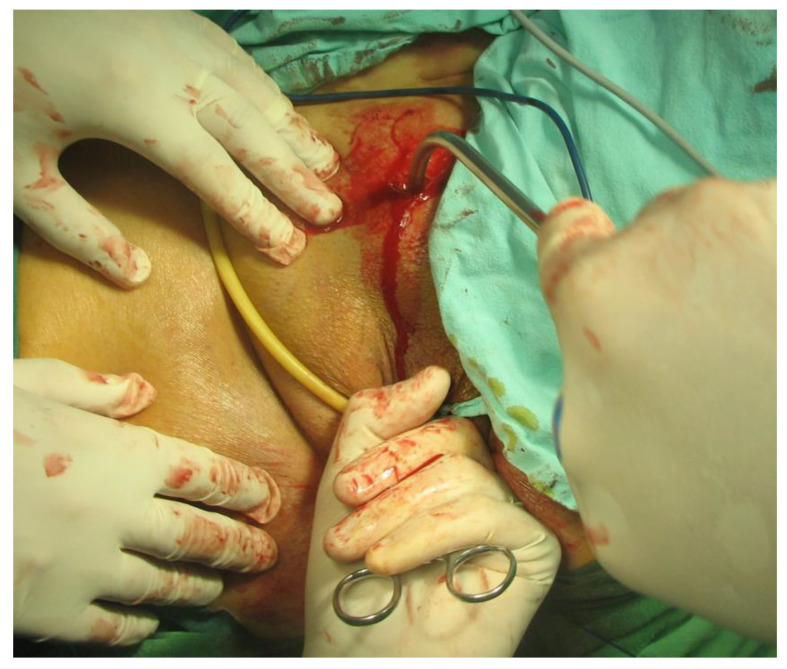
Suprapubic puncture using the ‘out-in’ needle placement technique for TVT. (Personal Collection).

**Figure 2:**
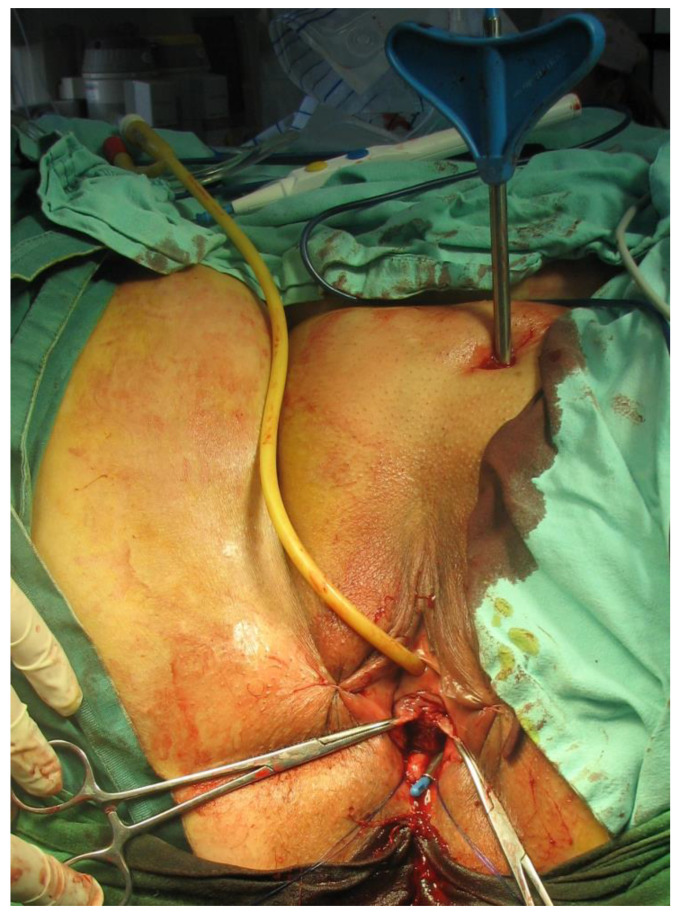
The TVT needle is positioned in the retropubic space and brought out transvaginally (Personal Collection).

**Figure 3:**
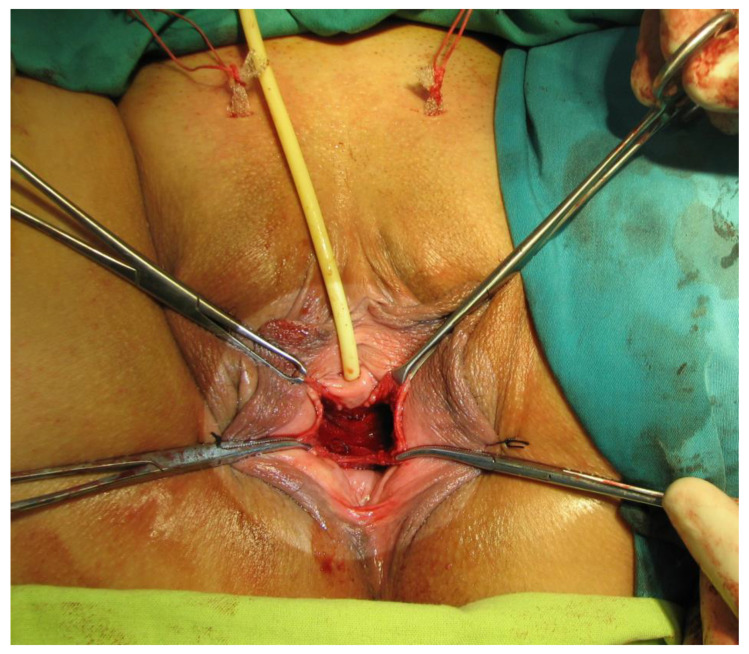
The polypropylene sling in its final suburethral position, with the ends exteriorized suprapubically (Personal Collection).

**Figure 4:**
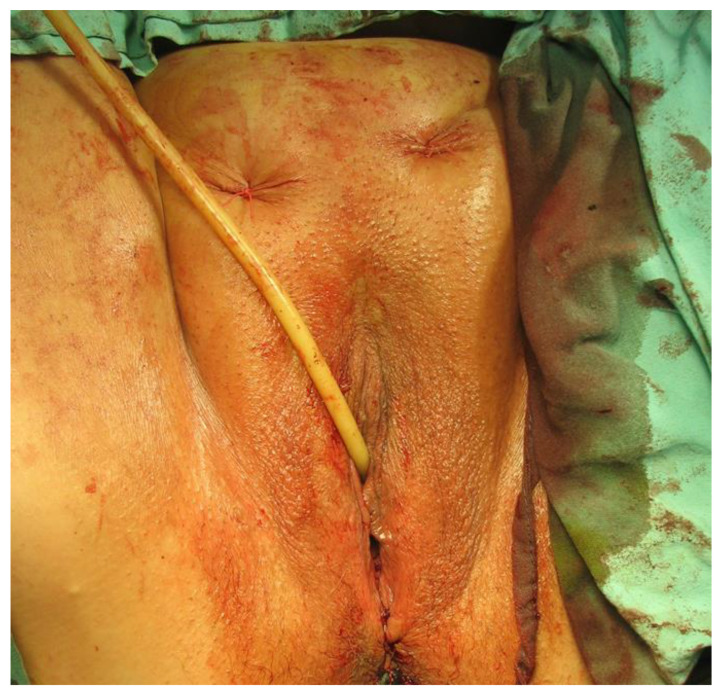
Final surgical outcome of the TVT technique (Personal Collection).

In the period 2010 to 2022, a total of 455 patients (p) underwent surgery for stress urinary incontinence in Sibiu county hospital’s urology department. In these surgeries, the transobturator technique (TOT) was applied to 353 patients (77.58%), and the retropubic TVT technique to 102 patients (22.42%). Surgical procedures were performed by the identical surgical team, who used internationally approved techniques. Some of the surgical steps are depicted in Figure 1, Figure 2, Figure 3 and Figure 4.

In this retrospective study, the inclusion criteria were being aged over 18 years-of-age, a positive diagnosis of stress urinary incontinence and informed consent. Those with incomplete data or unclear diagnoses were excluded from the study. The selection of each patient’s type of surgical intervention was done randomly.

Accurate diagnosis and appropriate surgical indication are crucial and were determined based on anamnesis (history of incontinence, bladder diary, physiological and pathological events), and a complete clinical examination, involving the examination of the abdomen and pelvis; vaginal and rectal swabs; incontinence provocation tests, namely theBonney test, Q Tip-test and Ulmsteen maneuver; paraclinical examinations; abdominal and vaginal ultrasound; and laboratory tests of urine, urine culture, renal function, blood count and coagulogram.

The prostheses used to perform the techniques were monofilament polypropylene, with a width of 1 cm and a length of 40 cm, with fixation without tension in the suburethral portion.

The operations were performed with spinal anesthesia or general anesthesia. Each patient received a single intraoperative dose of cephalosporin. The postoperative antibiotic regimen, if needed, was prescribed by the infectious disease physician. The position of the patients was lithotomy position, with a slight degree of Trendelenburg. The vaginal packing was removed from all patients on the first postoperative day, and the urethral catheter was removed on the second day. The postoperative evaluation of the patients involved clinical examination, ultrasound examination, urine examination and urine culture on day two, months three, six and twelve, and then annually. The postoperative follow-up period for the patients averaged 82.5 months. The follow-up duration was approximately 102 ± 30 months for tension-free vaginal tape (TVT) procedure patients, and 80.4 ± 13 months for transobturator tape (TOT) treatment counterparts. The follow-up protocol had several components: anamnesis, a micturition diary that patients filled out for seven days before their visit and a clinical examination. The clinical examination consisted of the cough test and Valsalva maneuver, which were both conducted in the supine position, along with abdominal ultrasound. Patient quality of life was also assessed by using specific questionnaires: the Urogenital Distress Inventory (UDI-6) and the Impact Incontinence Quality of Life (IIQ-7). In addition, the evaluation concentrated on tracking the degree of improvement of initial patient symptoms.

Intra- and postoperative complications were distributed according to the technique used. Postoperative complications were divided into immediate postoperative complications (first 48 h) and late postoperative complications, which were assessed in periodic evaluation visits.

In the identification of potential intraoperative complications, intraoperative ultrasound emerged as a crucial tool for the comprehensive assessment of pelvic organs, vascular structures and real-time issues, such as bleeding and hematoma formation. Ultrasound, in serving a dual purpose as an evaluative method and guidance system, played a pivotal role in ensuring real-time precision. In the postoperative context, additional imaging investigations, including CT and MRI, were used to meticulously detect potential complications, including bladder and ureteral injuries and hematomas. Cystoscopy retained its status as the preferred method for assessing bladder perforation in TVT placement, particularly when hematuria was present.

Categorical variables were presented (percentage %) and compared with Fischer’s exact test. Continuous variables were presented as a median with IQR and compared by the Mann–Whitney U test. A two-tailed significant *p*-value of 0.05 was then used to assess statistical significance, and statistical analysis was then conducted by using IBM SPSS version 22.0.

## 3. Results

In our retrospective study, which was conducted at our single center, we examined the outcomes of 455 women who had undergone surgical procedures to address stress incontinence, shedding light on the efficiency and safety profiles of two established techniques. Our findings indicated an overall complication rate of 3.51% in 16 cases. Complications were significantly more prevalent in the TVT group (Table 1), with bladder injury and retropubic hematoma emerging as complications with a higher incidence, raising important questions about the safety of this particular procedure (*p*-value = 0.001 and 0.011, respectively).

Bladder injuries were identified by using intraoperative cystoscopy and were promptly resolved by removing the tape and prolonged urethral catheter and replacing them. In contrast, bladder injuries occurred in three TOT group patients (0.84%), and these injuries were identified subsequent to hematuria onset being observed in the urethral catheter. These injuries were resolved through bladder suture using resorbable vicryl suture, which was implemented in a single layer. Despite vaginal injuries occurring exclusively in the TOT group, our study did not establish a statistically significant association between vaginal injury and either of the techniques (Table 1). Furthermore, bladder injuries were reported in the TVT group in three patients (2.94%) with a history of total hysterectomy (Table 2). In the TOT group, three patients (0.84%) presented bladder injuries after the previous implementation of the Burch procedure for stress urinary incontinence. The vaginal lesion was only registered in four TOT group patients (1.13%) with a previous history colporrhaphy. And retropubic hematoma was only found in TVT group member who had previously undergone a hysterectomy and who had a past history of colporrhaphy (Table 2).

In our analysis of complications in stress incontinence procedure patients, we did not find significant age differences among those experiencing complications from either TVT or TOT procedures (Table 3). Similarly, we did not find any association between surgical history and complications for either group. Notably, three TOT group patients with no previous surgical history suffered bladder injuries, prompting us to further investigate the unique factors that contributed to this. A significant finding also emerged from the consideration of obesity, when higher prevalence was observed in the TVT group experiencing complications, compared to the TOT counterpart (*p* = 0.013).

Vaginal lesions were exclusively documented in the TOT group, with an incidence of 1.13% (Table 1), and were found in the passage through the obturator fossa, with the device tip penetrating the vaginal side wall.

Retropubic hematoma occurrences were exclusively noted in patients undergoing the TVT procedure, and found to affect three individuals (2.94% incidence—Table 1). The cases of hematoma were effectively managed through conservative treatment, specifically hemostatic measures, analgesics and antibiotic therapy.

Immediate postoperative complications documented within the initial 48 h primarily included urinary retention, febrile syndrome and urinary tract infection. Overall, immediate postoperative complications were recorded in 23 patients (5.05%), more frequently in the TVT group (Table 4).

Acute urinary retention occurred more frequently in the TVT group, with an incidence of 13.72%, compared to 1.14% in the TOT counterpart. Urinary retention was observed following the removal of the urethral catheter, and this was addressed by reinserting and retaining the catheter for an additional five days (Table 4).

The rest of the patients who presented chronic postoperative urinary retention (five (4.9%) from the TVT group and two (0.57%) from the TOT counterpart), benefited from conservative treatment, specifically the administration of cholinomimetic medication (bromide of neostigmine 15 mg, three times a day; pyridostigmine bromide 60 mg, four times a day) and alpha-blocker (tamsulosin hydrochloride 0.4 mg, daily) for one month.

Febrile syndrome, another complication, was only present in one patient (0.98%) from the TVT group, who also presented a retropubic hematoma, but recovered after the administration of antibiotic, analgesics, antipyretics for seven days. Urinary infection also occurred in the TVT group in three patients (2.95%), who were treated with antibiotics, in accordance with the antibiogram, for sevendays.

The minimum follow-up period for the final patients was 18 months, much longer than 82.5 months, the average follow-up evaluation for the postoperative patients an average of 102 ± 30 months for the TVT group and 80.4 ± 13 months for the TOT counterpart). Late postoperative complications were documented in a total of 33 patients, in 20 TOT group members (5.66%), and 13 TVT group counterparts (7.84%) (Table 4).

The two techniques did not significantly differ in terms of the occurrence of vaginal erosion (Table 4), and in the instances when this complication was found, it was addressed by removing the mesh from the erosion site and using local anesthesia to perform secondary suturing of the vaginal lesion.

Recurrence of incontinence was documented in 13 TOT group patients (3.7%), and was resolved through surgical reintervention with spinal anesthesia that remounted the band, in a manner similar to the TVT procedure.

De novo urgency was more prevalent in the TVT group and affected individuals from the group received antimuscarinic treatment, specifically solifenacin, in a daily oral dose of 5 mg (Table 4).

In the follow-up period, we closely monitored the progress of initial symptoms, noting how both techniques demonstrated substantial improvement in pelvic pain, urgency and dyspareunia, and reflecting on the (crucial) finding that the TVT group’s improvement in incontinence was significantly more pronounced (Table 5).

In acknowledging the significant relevance of patients’ medical history, we propose to conduct an evaluation that considers how it can predict late postoperative complications (Table 6).

## 4. Discussion

It is undoubtedly true that establishing a precise diagnosis of the condition and being well-versed in all available treatment options are both of fundamental importance in the management of stress urinary incontinence patients (SUI) [1]. Moreover, it is essential for physicians to continually recognize the significant social and quality of life impacts this condition can have on patients. Patients themselves need to be fully informed about SUI, and aware of the possible complications associated with each surgical method [19]. Consequently, the physician, in partnership with the patient, should select the most appropriate surgical technique for the treatment of SUI. 

The introduction of polypropylene mesh tapes was a significant advance in the treatment of stress urinary incontinence (SUI) [13], which uses a synthetic mesh to construct a pubo-urethral neo-ligament, thereby enhancing urethral reinforcement. Although initially recommended for type 3 stress incontinence, recent studies have suggested it can be efficiently expanded to encompass all categories of the Blaivas classification for SUI [20,21,22].

The tension-free vaginal tape (TVT) has been extensively researched, and has been prominent in clinical studies for nearly 15 years. Ward and Hilton, in one (notable, multicenter and randomized) controlled trial, presented both the short-term and medium-term outcomes, comparing the efficacy of TVT to open colposuspension [23], describing an objective five-year cure rate of 81% in the TVT group, compared to the open colposuspension group. While this difference was not statistically significant, it suggests both procedures can provide a viable treatment. The TVT group had three cases of late pelvic erosion, and the open colposuspension group had higher rates of postoperative enterocele and cystocele [23]. This research offers clinical proof that supports the TVT method, suggesting it provides a similar level of efficacy to open colposuspension, but without entailing the complications associated with open surgery. More recent studies, conducted by Saida Abrar et al. across eight years, show that although TVT is considered to be the gold standard in many respects, surgeons must remember that colposuspension remains a valuable surgical technique, often offering more than satisfactory efficacy [24]. Moreover, low-tension suburethral bands have their own complications, such as urinary retention, de novo detrusor instability, infection and erosion, chronic pelvic pain, dyspareunia, which can all persist in the long term [25,26].

Our assessments of life quality showed no major differences between the groups at any point in the follow-up period, which aligns with Rechtberger et al, who undertook one of the most comprehensive and extensive randomized trials conducted to date. Their study of 537 patients, which compared retropubic and trans-obturator procedures over an 18-month follow-up period, reported no notable difference in clinical effectiveness, and found both techniques achieved a cure rate of around 75%. However, it is important to note that 6.5% of the surgeries that used the retropubic route were complicated by bladder perforation [27]. In our study, the retropubic TVT technique, bladder injury occurs in approximately 6% of cases, clearly contrasting with the TOT transobturator approach, which significantly reduces this risk to less than 1%. It is crucial to recognize that the majority of bladder injuries sustained in SUI surgeries are typically minor and do not adversely affect overall treatment outcomes. While the TOT method demonstrated a reduced occurrence of bladder injuries and instances of urinary urgency, it was associated with a more significant incidence of vaginal erosions. On the other hand, the TVT group exhibited a higher frequency of chronic urinary retention, necessitating additional surgical interventions in four patients. Furthermore, the TVT group also reported a larger number of cases with newly developed urgency symptoms that affected six patients, in contrast to only one TOT group patient. This comparison highlights the distinct postoperative complication profiles associated with both of our surgical methods, underscoring the importance of individualized patient assessment and management in choosing the appropriate surgical approach for stress urinary incontinence. The precise pathophysiology behind vaginal erosions caused by mesh materials in SUI surgeries does however remain somewhat elusive. Various predisposing factors have been identified, including conditions related to pelvic anatomy, such as genital atrophy, tissues with low estrogen levels, a history of previous vaginal surgeries or concurrent procedures, local infections and higher body weight [28]. In our study group, bladder erosions were recorded in six patients (1.31%); four patients (3.92%) in the TVT group, and two patients (0.56%) in the TOT group.

Fever remains a warning sign in surgical pathologies and should not be neglected, especially in patients who have been intubated, as they may develop ventilator-associated pneumonia [29]. In going beyond this pathology, we observe that while antibiotics are extremely helpful in both prophylaxis and treatment, they must be chosen with great care and in accordance with both local and international protocols, with the aim of avoiding the continually increasing resistance to them [30,31]. Excessive antibiotic therapy can also lead to dreaded complications such as Clostridioides difficile infection, which can have severely affect patient health and often leave microbiota transplantation as the only available option [32,33,34]. While future studies will certainly bring new methods of drug delivery with improved pharmacokinetics, which could reduce patient risks and reduce many complications [35]. Regardless of the chosen method, there were no significant differences in terms of fever syndrome onset and the safety profile of the two techniques was nearly identical in this regard.

Urinary urgency may affect as many as 20% of patients who undergo suburethral sling placement. A detailed review conducted in 2022 revealed a slight difference in the incidence of these symptoms when the transobturator and retropubic techniques are compared. This review suggests that while both methods are associated with the development of post-surgery urinary urgency, there are subtle variations in the frequency and severity of these symptoms that depend on the surgical approach used [36]. These findings from the literature correlate with our findings, indicating that although urinary urgency is a relatively common complication, there are no notable differences in incidence between the two types of interventions [28]. However, a study of 597 women with a five-year follow-up reported a higher incidence of urinary urgency in the TVT, compared with the TOT group, and also that urinary symptoms declined in both over time [37]. In our study, TOT showed higher alleviation of urgency symptoms (75% vs. 66%) although not at a statistically significant (*p* = 0.103), which justifies choosing TOT (rather than TVT) for patients presenting with urgency (48 vs. 3 patients enrolled). Also in patients presenting urgency symptoms, the TOT procedure shows a greater alleviation of urgency symptoms among all sling placement techniques [38].

A systematic review of the incidence of OAB (de novo overactive bladder) in sling placement procedures showed that the overall incidence of incontinence after midurethral sling procedures is about 9% (8.7% TOT vs. 9.8% TVTs) [39]. However, other studies showed little to no incontinence after comparing patients undergoing the TVT and TOT procedures (0% vs. 4.5%), which is identical with our own findings (0% vs. 3.7%) (Table 5) [40]. In this study it was suggested that TOT showing statistically significantly more unfavorable incontinence results could be due to the presence of urgency symptoms. Patients who present urgency or pelvic pain were statistically more likely to be treated with a transobturator approach (Table 5), despite the procedure being applied randomly. This could explain the occurrence of OAB after surgical treatment [39].

Pelvic pain is an important consideration in postoperative care, whose potential causes can range from hematoma formation to nerve damage. When employing the transobturator approach, injury to the adductor longus muscle might also be a source of discomfort, and so it is initially advisable to manage pain expectantly in the first week after the surgery. However, if the pain continues beyond this initial period, it becomes necessary to evaluate for more serious complications, such as nerve or muscle damage. Management typically involves using analgesics for pain relief. In cases where pain persists and becomes a significant issue, the consideration of surgical interventions, such as the excision of the problematic band, may be warranted. This approach underscores the need for careful monitoring and timely intervention to address complications that can potentially arise from pelvic surgeries [41,42,43]. Regardless of the technique employed, our study found pain management to be crucial, and the overall results obtained for alleviating pelvic pain were encouraging, in both techniques.

A review conducted in United Kingdom showed that patients with lower body mass index (BMI) (<30 kg/m^2^) were likely to report a well-being improvement, fewer incontinence symptoms and lower rates of worsening symptoms after MUS (mid-urethral-sling) [44]. It was expected that obesity would be a risk factor for long term complications. Moreover, our study shows that a BMI higher than 30 kg/m^2^ predicts the failure of MUS, when adjusted for presenting symptoms and procedure type. We could not find any surgical history relevant to the worst outcome (Table 6). In a study conducted in Finland, hysterectomy did not indicate the ineffectiveness of the procedure, regardless of the MUS procedure type. This was because the rate of reoperation following MUS did not vary, whether between groups that underwent different types of procedures after hysterectomy, or control groups that did not undergo hysterectomy [45], findings similar to those produced by this study. A study that evaluated the concomitant anterior colporrhaphy with MUS and MUS showed that the latter is more efficient, but not at a statistically significant level, which clearly resembles our finding that anterior colporrhaphy has a small influence on outcome. [46]

There are only a small number of studies that compare these two surgical techniques across a substantial patient population in the current literature. The primary focus of these studies has been on the identification and appropriate management of complications, both during and after surgery. However, the literature is currently limited by the absence of a consensus on the efficacy of these two techniques or a comparative analysis. This lack of agreement leads to contradictory conclusions in various studies, with some authors favoring one technique over the other on the basis of their findings [5,47,48,49]. One consistent and universal finding reproduced across these studies is that both techniques have shown clear evidence of safety and satisfactory efficacy, not just in the short term but also over medium and long-term periods [50,51]. In addition to their proven safety, each technique has a specific complication profile, enabling physicians to effectively anticipate and document any potential intraoperative and postoperative issues. It is also important to highlight that the “best” technique is often the one that is most familiar and most frequently implemented by the surgical team [52]. Therefore, if a surgical team is more comfortable with a particular method, it is likely that it will yield fewer complications. However, the present study provides concrete results that will contribute to outlining a profile for each technique, helping the surgeon to anticipate, based on the chosen technique, the stage of the treatment process at which more complications. This will then enable them to take preventive measures, regardless of their level of experience.

## 5. Conclusions

Tension-free vaginal tape (TVT) and transobturator tape (TOT) both stand out as highly efficacious methodologies for addressing stress urinary incontinence. Beyond efficiency, their safety profile is sufficiently strong to enable and justify their application to a wide range of patients. On the other hand, obesity has been identified as a significant predictor of failure, and it must accordingly be taken into account.

The choice of the optimal treatment method depends on several factors, of which the most important are the experience of the attending physician, and the patient’s profile and desire. In the area of long-term outcomes, TVT has shown positive results, highlighting its modest superiority over TOT in this respect. And while TVT remains the method with the most intraoperative complications, this should be balanced against the fact that, in the long term, the postoperative complications of the two methods are comparable.

### Study Limitations and Strengths

This study has notable strengths, including a substantial patient cohort, the consistent involvement of the same surgical team, an extended follow-up duration, and a meticulous and promptly assessed evaluation of complications. The significant number of patients enhances the statistical robustness of findings, and the continuity of the operating team ensures consistency in procedural techniques and care. The long follow-up period contributes to a comprehensive understanding of outcomes, and the detailed tracking and prompt assessment of complications highlights the study’s methodological rigor.

However, certain weaknesses should be acknowledged. The single-center nature of the study and its application within a relatively confined geographical area, both limit the broad generalizability of the results to diverse populations with different characteristics. The study’s limited scope suggests that caution must be exercised when seeking to extrapolate the findings to other demographic groups.

## Figures and Tables

**Table 1:** Intraoperative complications.

Intraoperative Complications	TOT (353 p)	TVT (102 p)	*p*-Value
Overall complications	7 (1.98%)	9 (8.82%)	0.0009 *
Bladder injury	3 (0.84%)	6 (5.88%)	0.001 *
Vaginal injury	4 (1.13%)	0	0.953
Retropubic hematoma	0	3 (2.94%)	0.011 *

* Significant values are flagged.

**Table 2:** Complications and surgical history.

History of Urogynecology Surgery	*N*	Bladder Injury		Vaginal Injury		Retropubic Hematoma	
		TVT *N* = 6	TOT *N* = 3	TVT *N* = 0	TOT *N* = 4	TVT *N* = 3	TOT *N* = 0
Hysterectomy	101	3 (2.94%)	0	0	0	2 (1.96%)	0
Anterior colporrhaphy	32	0	0	0	4 (1.13%)	1 (0.98%)	0
Posterior colporrhapy	22	0	0	0	0	0	0
Burch procedure	11	0	3 (0.84%)	0	0	0	0
No history	289	3 (2.94%)	0	0	0	0	0

**Table 3:** Considering complications on the basis of demographics and clinical history.

Variable	TOT (*n* = 7)	TVT (*n* = 9)	*p*-Value
Age (years)	66 (64–67.5)	67 (66–69)	0.368
Obesity	2 (28%)	8 (88%)	0.013 *
Clinical History			
Hysterectomy	0	4 (44%)	0.307
Anterior Colporraphy	4 (57%)	2 (22%)	0.157
Burch Procedure	3 (42%)	0	0.072
No history	0	3(33%)	0.401

* significant values are flagged.

**Table 4:** Immediate and late postoperative complications.

Variable	TVT (102 p)	TOT (353 p)	*p*-Value
Immediate postoperative complications			
Overall	18 (17.64%)	5 (1.41%)	<0.001 *
Acute retention of urine	14 (13.72%)	5 (1.41%)	<0.001 *
Febrile syndrome	1 (0.98%)	0 (0%)	0.968
Urinary tract infection	3 (2.95%)	0 (0%)	0.687
Late postoperative complications			
Overall	13 (12.74%)	20 (5.66%)	0.0001 *
Vaginal erosion	2 (1.96%)	4 (1.13%)	0.618
Recurrence of incontinence	0 (0%)	13 (3.7%)	0.047 *
De novo urgency	6 (5.88%)	1 (0.28%)	0.0006 *
Chronic retention of urine	5 (4.90%)	2 (0.57%)	0.0002 *

* Significant values are flagged.

**Table 5:** Postoperative symptom alleviation.

Variable	TVT	TOT	*p*-Value
Preoperative symptom			
Urgency	3 (2.9%)	48 (13.5%)	0.002 *
Pelvic pain	6 (5.8%)	91 (25.6%)	0.0001 *
Dispareunia	7 (6.8%)	37 (10.4%)	0.282
Postoperative symptom alleviation			
Incontinence	102 (100%)	340 (96.3%)	0.047 *
Urgency	2 (66%)	36 (75%)	0.103
Pelvic pain	5 (83.3%)	81(89.01%)	0.180
Dispareunia	5 (71.42%)	33 (89.18%)	0.209

* Significant values are flagged.

**Table 6:** Univariable and multivariable regression for predictors of late postoperative complications.

Variable	Univariable Logistic Regression				Multivariable Logistic Regression			
	Odd Ratio	*p* Value	CI of 95%		Odd Ratio	*p*-Value	CI of 95%	
Hysterectomy	1.895	0.785	0.456	3.124	1.452	0.825	0.402	3.125
Burch procedure	8.856	0.072	0.986	34.123	6.145	0.096	0.953	28.124
Age	1.122	0.132	0.913	1.225	1.023	0.175	0.894	1.329
Obesity *	1.862	0.002 *	1.356	2.102	1.125	0.037 *	1.105	1.533
Anterior colporrhaphy	7.758	0.096	0.975	31.125	5.566	0.102	0.932	25.223
Posterior colporrhaphy	0.356	0.956	0.015	0.452	0.326	0.988	0.014	0.325

* significant values are flagged.

## Data Availability

The datasets generated and analyzed in this study are not publicly available because of institutional restrictions. However, the corresponding author will provide them, upon reasonable request.
